# Psychological and physical impact of wearing personal protective equipment among health care workers during the COVID-19 pandemic

**DOI:** 10.6026/973206300200822

**Published:** 2024-08-31

**Authors:** Madhavrao Chavan, Sushil Sharma, Santenna Chenchula, Gaurav Rangari, Arup Kumar Misra, Pavani Saggurthi

**Affiliations:** 1 Department of Pharmacology, All India Institute of Medical Sciences, Mangalagiri, Andhra Pradesh, India

**Keywords:** COVID-19, PPE, health care workers, psychological effects, physical effects

## Abstract

Healthcare workers (HCWs) are front-line warriors in the battle against the COVID-19 pandemic. Therefore, it is of interest to assess the psychological and physical effects of personal protective equipment (PPE) among HCWs caring for COVID-19 patients. This cross-sectional study utilized a validated, well-structured questionnaire consisting of 24 questions to collect information on the psychological and physical effects experienced by HCWs. The study adhered to relevant EQUATOR guidelines for reporting. The study collected online data from 114 HCWs working in COVID-19 settings while using PPE. Among the respondents, 33% reported anxiety, 23.1% experienced apprehension after donning PPE, and 68.1% felt discomfort. Additionally, 46.2% required up to 12 hours for psychological restoration after a COVID-19 duty shift, 17.6% were worried about the risk of infection, and 28.6% were extremely worried about infecting family members while on duty. The findings highlight significant discomfort, anxiety, and apprehension among HCWs due to prolonged PPE use, reflecting the immense psychological burden of working in high-risk environments during the pandemic. These results emphasize the need for comprehensive support systems and interventions to address the multifaceted needs of HCWs, including psychological support, adequate breaks, and measures to mitigate physical discomfort.

## Background:

Healthcare workers (HCWs), are part of the front-line warriors in this on-going battle against the novel coronavirus disease 2019 (nCOVID-19), a highly infectious disease caused by SARS-CoV-2 virus [1]. To date, millions of HCWs have been infected with SARS-CoV-2 and lost their lives during the battle [2]. Personal protective equipment (PPE) kits help to prevent contamination of healthcare workers from highly contagious infections such as SARS and Ebola virus disease [3]. The PPE kit includes protective clothing, gloves, masks, and goggles head and show cover [3]. The frequency of adverse events among HCWs due to PPE use is considerable [3]. The Centre for Disease Control (CDC) donning and doffing procedures, such as one-step glove and gown removal, double-gloving, spoken instructions during doffing, and using glove disinfection, may reduce contamination and increase compliance [4]. Maximum covering of the body with PPE and a higher-level specification of masks and respirators are associated with better protection it offers [5- 6]. Previous studies have shown that more active training in PPE use may reduce PPE and doffing errors more than passive training, and front-line HCWs need an appropriate provision of PPE, training in its use, and comprehensive and consistent guidance to protect HCWs from COVID-19 infection [7-8]. However, findings from Wuhan, China, where COVID-19 spread across the world, showed that wearing a PPE kit while on COVID-19 duty affected HCWs psychologically and physically [9, 10, 11-12]. Working for a longer duration by wearing PPE, and under work, pressure was associated with psychological and mental illness among HCWs [11, 13-14]. Therefore, it is of interest to study the psychological and physical effects on HCWs on COVID-19 duty associated with prolonged wearing of PPE and its correlation with demographic parameters such as sex, age, occupation, COVID-19 work area, PPE supply and accessibility and the sufficiency of PPE training provided in their workplace, working time, vaccination status, and apprehension of risk of exposure to COVID-19 at work by self and family members.

## Methods:

The present study was a cross-sectional questionnaire-based descriptive study. As there is no validated survey in the literature, a survey tool was developed in Google Form for the present study based on a literature review that identified common PPE-related side effects experienced by HCWs, both physical and psychological effects, and was validated with 30 HCWs across the world, during the COVID-19 pandemic (Annexure 1 Table S1) [9-14]. We adhered to relevant EQUATOR guidelines to report (Annexure 2 STROBE Statement).

The final survey contains three sections that comprise yes or no questions, a few open-ended questions, and questions answered with a Likert scale ranging from '1' (slight) to '5' (Extreme). Study participants must respond to all 24 questions for the response form to be submitted. Demographic data such as gender and age, occupation title, location of COVID-19 duty, vaccination status, history of infection with SARS-CoV-2 during the COVID-19 duty, history of allergic reactions to the PPE, bronchial asthma, training on universal biosafety measures before the COVID-19 duty, supply and accessibility of an appropriately sized PPE at duty location, experience on SOPs regarding the Donning and Doffing of a PPE, State of mind after donning a PPE, the maximum time spent wearing a PPE in one COVID-19 duty shift, duration of time that they become uncomfortable after wearing a PPE, and how much confidence in protection from wearing a PPE during the COVID-19 duty. In addition, participants were also asked about their discomfort while wearing PPE during COVID-19 duty (dizziness or palpitation, suffocation/breathlessness, retro auricular pain, micturition desire, nausea or vomiting, thirst or dry throat, inconvenience at work, nose pain, fogg/mist on goggles/face shield, drenched with sweating, increased body heat, exhausted), cutaneous adverse effects from wearing PPE (skin rashes, redness, itching/eczema, dryness of the skin, skin erosion/pressure sores, aggravation of acne, no skin complaints), duration of time needed to restore physically after a COVID-19 duty shift (<12 hours to >48 hours), the time needed to restore psychologically after a COVID-19 duty shift (<12 hours to >48 hours), worry about the risk of getting infected while on COVID-19 duty hours even after wearing PPE and worry about the risk of infecting family members while on COVID duty hours, even after wearing PPE in their workplace, were collected. After obtaining institutional ethical committee (IEC) approval, data were collected by sending a survey Google form to all healthcare workers working in the COVID-19 area across the world. There were 24 questions, and among them, 3 had multiple-response options. The maximum time to complete the questionnaire was 10 minutes. Participants were asked to voluntarily give their consent to participate in the present online survey study. We obtained e-informed consent to participate in the study, with no incentives offered.

## Statistical analysis:

All statistical analyses were carried out using SPSS software. Descriptive data are presented as frequencies, percentages and numbers. Physical and psychological adverse effects associated with PPE were reported as numbers and percentages, and the chi-square test or Fisher exact test was used for intergroup comparisons (sex, occupation, age, workplace, department, and duration of PPE wearing), and the data are presented in tables and graphs. *P values* less than 0.05 were considered statistically significant. Data were collected, tabulated, and analysed with MS Excel and SPSS 25.0 software. Data are presented in percentages, bar and pie diagrams, and proportions.

## Results:

## Demographic characteristics:

A total of 114 HCWs who wore PPE kits while on COVID-19 duty participated in the survey. In the present study, the majority of respondent HCWs were nurses (52.8%), physicians (30.8%), technicians (2.2%), and other healthcare workers (13.2%). Among all the responders, 54.9% were females and 45.1% were males. Among the study participants, 54.9% were aged between 20-30 years, 38.5% were aged 31-40 years, and only 1.1% were aged >50 years. Among all respondents, the majority performed duties in the isolation ward (58.2%), followed by the ICU (35.5%) and clinical laboratories (3.3%). [Supplementary file: Annexure 2: Table S2: Demographic Parameters of the Study Participants; Table S3 study questionnaire parameters with the percentage of response; Table S4: Age wise, occupation wise and gender wise analysis].

## Vaccination status:

Among the study participants, 57.1% were vaccinated and 42.9% were unvaccinated when performing COVID-19 duties. Among the vaccinated population, 52.7% received two doses, 7.7% received only a single dose of the COVID-19 vaccine, and 39.6% of HCWs were unvaccinated.

## Infection with SARS-CoV-2:

Among the study participants, 36.3% of HCWs were infected with COVID-19 while on a COVID-19 duty.

## Availability and training on PPE for COVID-19 HCWs:

Only 54.9% of HCWs had received training on universal biosafety measures before the COVID-19 duty. At the COVID-19 duty location, a total of 67% were provided with the proper size of PPE, while 33% reported improperly sized PPE. A well-acquainted SOP including donning and doffing a PPE kit was reported as very well by 35.2% and not well acquainted by 4.4% of HCWs. The maximum time spent wearing PPE in one COVID-19 duty shift was 4-6 hours (37.4%) and 6-8 hours (3.3%).

## Psychological effects of PPE:

Among study respondent HCWs, 68.1% reported discomfort, 33% as anxiety, 23.1% as apprehension and 17.6% scared as the state of mind after donning PPE. A total of 30.8% of HCWs reported that in < 1 hour, they were uncomfortable after wearing the PPE, 28.6% of HCWs in 1 to 2 hours, 25.3% of HCWs in 2 to 4 hours, and only 1.1% of HCWs in 8 to 12 hours. Confidence in protection by wearing PPE was reported as extremely confident by 15.8%, very confident by only 4%, fairly confident by 33%, and not confident by 2.2% of HCWs. For time needed for psychological restoration after a COVID-19 duty shift, at least ≤12 hours by 46.2%, 12-24 hours by 26.4%, 25-36 hours by 4.4%, 37-48 hours by 4.4%, > 48 hours by 13.2% and no time needed by only 5.5%. During the COVID-19 duty hours, HCWs worried about the risk of becoming infected, with 27.5% being slightly worried, 19.8% being somewhat worried, 17.6% being fairly and/or very much worried, and 11.6% being extremely worried even after wearing PPE. Study respondent HCWs also reported their worry about the risk of infecting family members while on COVID duty hours, as they were extremely worried by 28.6%, very worried by 22%, and fairly worried by 13.2%, and only 6.6% of HCWs reported never worried (Figure 2).

## Physical effects of PPE:

**Figure 1:**
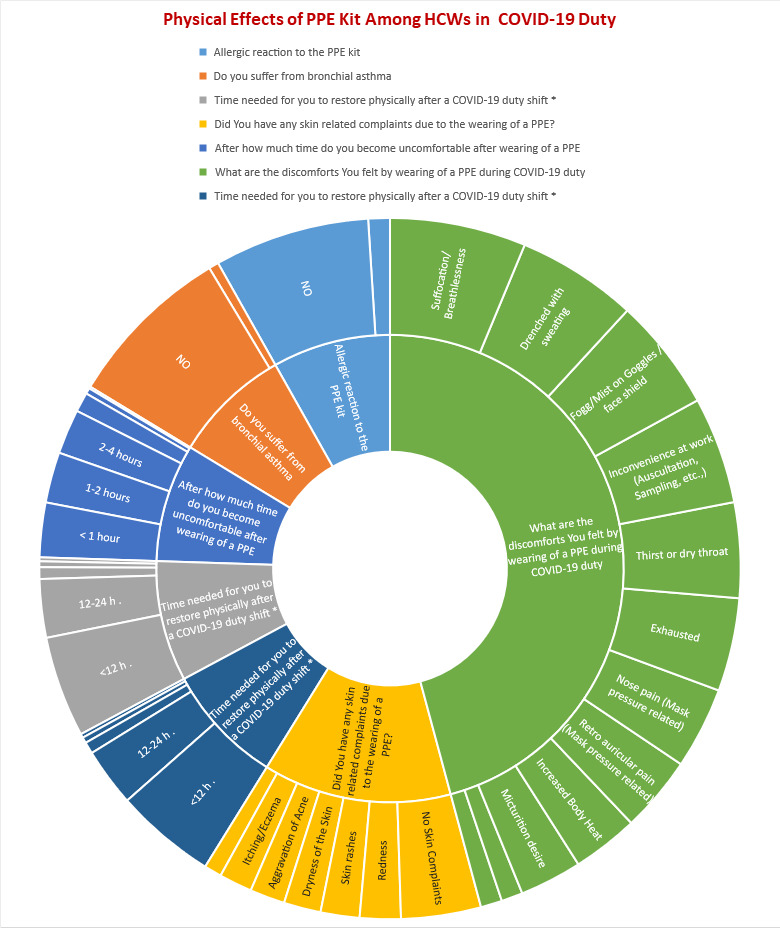
Physical effects of PPE among HCWs on COVID-19 duty

**Figure 2:**
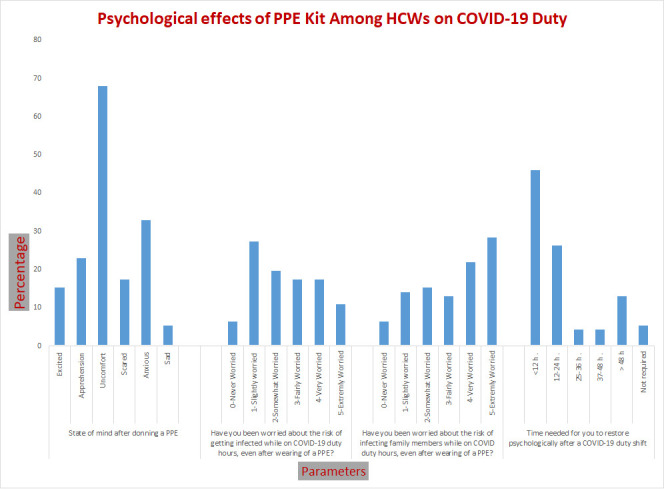
Psychological effects of PPE among HCWs on COVID-19 duty

A history of allergic reaction to the PPE kit was reported by 12.2% of HCWs, and bronchial asthma was reported by 5.6% of HCWs. Discomforts felt by wearing PPE while doing COVID-19 duties were reported as suffocation/breathlessness by 76.9%, retro auricular pain by 42.9%, micturition desire by 35.2%, nausea/vomiting by 12.1%, thirst or dry throat by 53.8%, inconvenience at work such as auscultation, sampling, etc., by 60.4%, nose pain by 46.2%, fog/mist on goggles/face shield by 63.7%, drenched with sweating by 68.1%, increased body heat by 37.4%, exhaust by 52.7%, and dizziness or palpitation by 12.1%. Several skin-related complaints due to the wearing of PPE were reported by HCWs, such as 23.1% reported redness, 22% reported skin rashes, 20.9% reported dryness of the skin, 19.8% reported aggravation of acne, 18.7% reported itching/eczema, 9.9% reported skin erosion/pressure sores and 45.1% of HCWs not reported any skin complaints. In the present study, the majority of HCWs (57.1%) reported requiring more than 12 hours to restore physically after a COVID-19 duty shift, 12-24 hours by 33%, 25-36 hours by 3.3%, > 48 hours by 6.6% and no time needed by only 2.2% of HCWs (Figure 1).

## Discussion:

The present study explores the physical and psychological effects of PPE kits experienced by HCWs; our findings add many critical issues regarding PPE among HCWs. The Government of India confirmed India's first case of COVID-19 on 30 January 2020 in the state of Kerala, when a university student from Wuhan travelled back to the state [15]. However, from March 20, 2020 onwards, there was exponential growth in the daily number of COVID-19 cases at the pan-India level [15]. Since then, all HCWs, as front-line workers, have been battling SARS-CoV-2 by wearing PPE as a protective measure. However, all frontline HCWs encountered several physical and psychological problems at varying levels as a result of wearing PPE [16]. According to the present questionnaire survey, there was a high prevalence of uncomfortable symptoms suffered by the HCWs during their fight against the COVID-19 epidemic, although active and timely training was helpful for the effective prevention of infection. More complaints of discomfort followed by anxiety and apprehension were reported by physicians, nurses, technicians and other HCWs working at a COVID-19-designated hospital. The vaccination status of the HCWs who participated in the present study was very low, and among the vaccinated HCWs, half were double vaccinated at the time of the study. In India, COVID-19 vaccines have been available since January 2021. Vaccination against COVID-19 prevents the spread of COVID-19 and decreases the severity of infection [17-18]. According to the results of the intergroup comparison in the present study, nurses and physicians are vaccinated more than other HCWs while doing COVID-19 duties.

Training on nosocomial infection before treating patients in wards is of considerable significance for preventing HCWs from contracting COVID-19 [17]. Only half of the study participants reported receiving proper training on universal biosafety measures before the COVID-19 duty. A fair number of participants reported receiving a proper amount of PPE; however, a very smaller number of participants reported being extremely well and very well acquainted with the SOPs regarding the Donning and Doffing of PPE. All HCWs should be provided with PPE of proper size and need very well-acquainted with the correct procedure for donning and doffing the PPE [19]. Regular interactive training on the prevention of nosocomial infection and the SOP for wearing PPE can considerably reduce the risk of HCWs' exposure to COVID-19 [19]. In the present study, the majority of HCWs reported discomfort within one hour with symptoms such as suffocation, retro auricular pain, micturition desire, nausea or vomiting, thirst or dry throat, inconvenience at work such as auscultation, sampling, nose pain, fogg/mist on goggles/face shield, drenched with sweating, increased body heat by, exhaust and dizziness or palpitations. In addition, there were several skin-related complaints, such as redness, skin rashes, and dryness of the skin, aggravation of acne, itching /eczema, and skin erosion/pressure sores. A systematic review of 16 articles from 6 different countries estimated the pooled prevalence of physical psychological effects of wearing PPE as follows: skin lesions (47-66%), headache (37-64%), sweating (56-90%), breathing difficulty (23-68%), vision difficulty (21-94%), thirst/dry mouth (30-77%), fatigue (58-76%), communication difficulty (47-94%), anxiety (24-33%), and fear (10-17%) [16].

To prevent discomfort related to wearing PPE, some of the essential measures, such as using moisturizers before putting on and after taking off gloves, wearing a properly fitted mask and applying moisturizer or gel beforehand for lubrication, non-irritating products for hand-washing, applying adhesive bandages on the portions of the skin in contact with the mask to help reduce friction and applying anti-mist agents on the goggles to prevent misting, are essential measures that help in preventing PPE-related discomfort [20, 21-23]. According to the present study, the majority of HCWs reported needing more than 12 hours to restore physically after a COVID-19 duty shift. Hence, a 24-hour break between shifts is recommended for HCWs to restore them physically from fatigue and work pressure [16]. However, even a 12-to-18-hour break between shifts might also be beneficial [16].

During the COVID-19 duty hours, HCWs worried about the risk of becoming infected even after wearing PPE. In addition, HCWs also reported their worry about the risk of infecting family members during COVID-19 duty hours. Hence, timely psychological interventions that build confidence and relieve stress are important considerations [24]. According to a survey on HCWs' emotional problems and coping strategies, positive attitudes in the workplace, clinical improvement of infected colleagues, and halting disease transmission among HCWs after adopting strict protective measures alleviated their fear and supported them through the pandemic [25]. For psychological restoration after a COVID-19 duty shift, the majority of patients reported at least 12 hours. Thus, a rational focus on facts and timely psychological assistance, such as offering coping strategies and measures to provide adequate medical equipment to treat patients and prevent HCW infection, is beneficial. Although several drugs have been repurposed as antivirals against COVID-19, to date, there are no effective drugs available [26-27]. Hence, in this on-going pandemic with the highly infectious and mutating SARS-CoV-2 era, all HCWS should be vaccinated, and regular preventive strategies against PPE-related physical and psychological effects must be followed [16-18, 28, 29, 30- 31]. This study has several limitations. The sampling was voluntary and online-based, creating possible selection bias. As a cross-sectional survey, no causation can be inferred.

## Conclusions:

Our study highlights the significant psychological and physical challenges healthcare workers face when wearing PPE while caring for COVID-19 patients, including widespread discomfort, anxiety, and apprehension. A substantial number of healthcare workers reported physical discomforts such as suffocation, sweating, and skin irritation, emphasizing the difficulties of prolonged PPE usage. These findings underscore the need for comprehensive support systems, including psychological assistance and measures to alleviate physical discomfort, to better support healthcare workers during the COVID-19 pandemic.

## Consent for publication:

Not Applicable

## Availability of data and materials:

Data available from the corresponding author upon reasonable request.

## Funding:

None to declare

## Ethics approval and consent to participate:

The study was approved by the Institutional Ethics Committee of the AIIMS Mangalagiri ((IEC: 2021-22/119) and all methods were carried out in accordance with relevant guidelines and regulations in the Declaration of Helsinki. Informed consent was obtained from the study participants.

## Authors' contributions:

All authors meet the ICMJE criteria for authorship. SC, MC, and SS lead the design and conceptualisation of the study. SC, MC, PS done the study analysis. SC drafted the manuscript, which GR, AK, and PS revised. All authors have reviewed and approved the final manuscript for publication.
